# Q&A: ChIP-seq technologies and the study of gene regulation

**DOI:** 10.1186/1741-7007-8-56

**Published:** 2010-05-14

**Authors:** Edison T Liu, Sebastian Pott, Mikael Huss

**Affiliations:** 1 Genome Institute of Singapore, 60 Biopolis Street, Number 02-01, Genome, Singapore 138672

## What is ChIP-seq?

ChIP-seq is short for chromatin immunoprecipitation-sequencing. Fundamentally, ChIP-seq is the sequencing of the genomic DNA fragments that co-precipitate with a DNA-binding protein that is under study. The DNA-binding proteins most frequently investigated in this way are transcription factors (for example, p53 or NFκB), chromatin-modifying enzymes (for example, p300, histone deacetylases), modified histones interacting with genomic DNA (for example, histone 3 trimethylated on lysine 4), and components of the basal transcriptional machinery (for example, RNA polymerase II). Theoretically, this technology can identify, in an unbiased manner, all DNA segments in the genome physically associated with a specific DNA-binding protein. We say 'unbiased' because whatever DNA comes down in the immunoprecipitate will be sequenced, and thus the technique does not rely on prior knowledge of precise DNA binding sites.

## What can I learn by knowing the DNA binding sites of proteins such as transcription factors?

Quite a bit. The major function of a transcription factor is to recognize and bind to specific sites in the genome, to recruit cofactors, and thus to regulate transcription. The first action of a transcription factor is to find and to bind DNA segments and ChIP-seq allows the binding sites of transcription factors to be identified across entire genomes. The DNA sequence motif that is recognized by the binding protein can be computed; the precise regulatory sites in the genome for any transcription factor can be identified; the direct downstream targets of any transcription factor can be determined; and the clustering of transcription-regulatory proteins at specific DNA sites can be assessed.

## How is it done?

**Figure 1:**
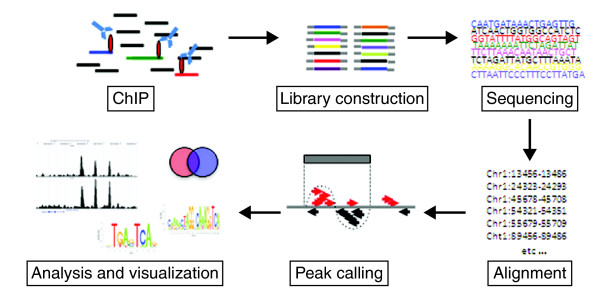
**Flow scheme of the central steps in the ChIP-seq procedure**.

The first step depends on the proteins under investigation (Figure 1). For many protein-DNA interactions, particularly for transiently bound factors, the first step might be to fix the interaction using formaldehyde as a cross-linker. This may not be necessary, however, for localizing histone modifications or for simply determining nucleosome positioning, because the histone-DNA interactions are generally strong enough to be maintained without using a cross-linking agent, and in this case a native ChIP (n-ChIP) without cross-linking might be preferable [1]. In the case of chromatin-remodeling enzymes such as histone deacetylases (HDACs) or histone acetyltransferases (HATs), an additional cross-linking step (using disuccinimidyl glutarate) can be included, to preserve protein-protein complexes before cross-linking with formaldehyde [2]. After cross-linking, the chromatin is fragmented into pieces of about 150 to 500 bp. For ChIP of transcription factors and under cross-linked conditions this is done using sonication. It is important to achieve sufficient and reproducible fragmentation, as preparation of the subsequent library of fragments for sequencing requires fragment sizes of 200 to 300 bp. In the case of n-ChIP, the DNA is digested with micrococcal nuclease to give a slightly better resolution, as it will leave the nucleosome as the smallest unit (approximately 150 bp).

After fragmentation, the next step is immunoprecipitation, using a specific antibody against the protein of interest. The success of a ChIP-seq project depends crucially on strong enrichment of the chromatin specifically bound by the protein under study. We routinely test a number of antibodies and choose the one with consistently high enrichment of DNA at a known binding site when compared with the DNA immunoprecipitated by a nonspecific control antibody such as anti-IgG and no enrichment at negative control sites.

Once the enrichment is convincing, the material is ready to be sequenced. If the amount of material is not a limiting factor (for example, when it comes from a tissue culture) the amount of DNA used for library preparation is about 10 to 15 ng. If the sequencing platform requires the incorporation of linkers and involves a PCR amplification step, this can be a considerable source of bias [3,4], and it is advisable to keep the number of cycles as low as possible. Once the material is amplified, DNA fragments of 200 to 300 bp long are selected and sequenced. Cross-contamination is a risk, both before PCR and afterwards, but can be minimized by preparing only a very small number of libraries in parallel and using separate gels when purifying the amplified libraries.

When material is limited, which is often the case with primary cell or tissue samples, smaller starting amounts of DNA have to be used. This is usually at the cost of additional rounds of amplification, which introduces amplification biases. However, one way of avoiding this might be to use the Helicos next-generation single-molecule sequencing platform, which can generate a sequencing library from 50 pg of starting material without requiring amplification [4].

Finally, the short sequenced fragments (known as tags) are computationally mapped by alignment to a reference genome and regions of enriched tag counts are identified, a step known as peak-calling.

## Why is ChIP-seq better than older approaches to finding DNA binding sites?

ChIP itself has been around for a while. This is where a DNA-binding protein is immunoprecipitated with its cognate DNA and the presence of DNA binding at a specific site is assessed by quantitative PCR. The problem with this approach is that only predetermined individual sites of known sequence can be studied.

An alternative technique that overcomes this limitation is DAM-ID, in which the protein of interest is fused to the *Escherichia coli *DNA adenine methyltransferase (DAM). When this fusion protein is expressed in cells, the adenines in the DNA adjacent to its binding site will be methylated. These sites can then be identified by methylation-sensitive restriction endonuclease mapping. But this technique is cumbersome, and requires overexpressing an artificial construct, limiting analysis to transfectable cell lines.

**Figure 2:**
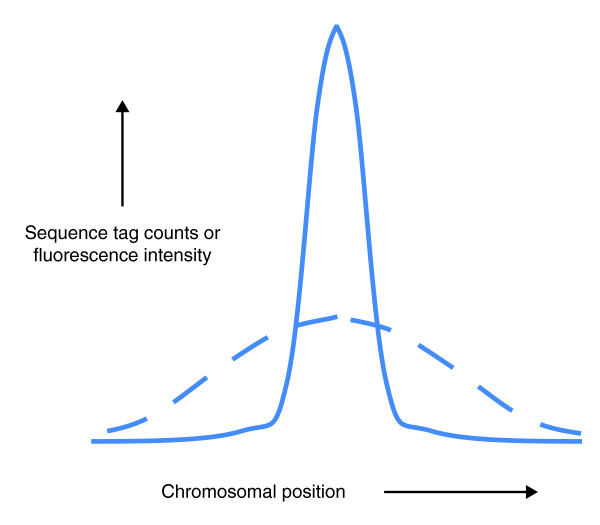
**Comparison of ChIP-seq and ChIP-chip**. Representative signals from ChIP-seq (solid line) and ChIP-chip (dashed line) show both greater dynamic range and higher resolution with ChIP-seq. Whereas three binding peaks are identified using ChIP-seq, only one broad peak is detected using ChIP-chip.

These problems are avoided in ChIP-chip, in which ChIP is coupled to DNA hybridization array (chip) technology. The DNA bound by the protein of interest is hybridized to a DNA microarray with probes that cover either the entire genome, or specific portions of the genome (for example, promoter regions). This is the closest methodology to ChIP-seq, but its mapping precision is lower, and the dynamic range of the readout is significantly less. The resolution and sensitivity of the two techniques are compared in Figure 2. Moreover, all hybridization approaches mask repetitive sequences. We have found that a significant portion (between 10 and 30%) of functional transcription factor binding sites are within repeats and are lost when ChIP-chip is used [5]. However, we still use ChIP-chip with custom arrays when specific binding sites are to be interrogated repeatedly over many experimental conditions.

## What are the technical problems with ChIP-seq?

Roughly speaking, ChIP-seq has three key steps that determine its success. The first and most crucial is antibody selection; the second is the actual sequencing, which is subject to several possible biases; and the third is the algorithmic analysis, including mapping and peak-calling.

The first requirement, obviously, is that the antibody has some specificity for the protein under study: this can be tested using a panel of recombinant proteins or cell lines transfected with different protein targets. Then, the antibody must be able to immunoprecipitate the target protein. Not all antibodies immunoprecipitate, and even when they do, they may not do well in ChIP. Ideally, earlier studies will have identified genomic sites where the protein is known to bind, and these sites can be used to optimize the ChIP conditions.

The second issue is sequencing, which is a 'black box' for many biologists, who are familiar with what goes in and what comes out, but perhaps not with the possible biases introduced in between. Next-generation sequencing approaches require bulk processing of DNA fragments and massively parallel sequencing. This means that even the slightest bias in the ligation of linkers, in PCR amplification, or in hybridization might result in some platform-dependent biases in the population data emerging from 10 million or more reads. The technologies are still evolving and the different formats have different biases. For this reason, it is important in a ChIP-seq experiment to run a control using 'input DNA' (non-ChIP genomic DNA) so that sequencing biases can be identified and adjusted for.

The third issue is mapping, which with short tags (around 25 to 35 bp) can be ambiguous in regions of high homology or in repeat regions. As the tag sequences get longer, this is less of a problem, but base calling and sequencing errors then limit the mappability. It is not uncommon to have only 50% of the reads mappable, though with more 'intelligent' mapping algorithms that take into account sequencing errors or polymorphisms, mappability has increased significantly. In ChIP-seq, the density of mapped sequence tags is a prime determinant of success. Illumina's ELAND algorithm and the MAQ (Mapping and Assembly with Quality) used to be the short-read mappers of choice, but a new generation of more efficient programs such as Bowtie, BWA (Burrows-Wheeler Alignment Tool) and BFAST (Blat-like Fast Accurate Search Tool) are gradually superseding them.

## That leaves peak-calling - how is that done?

There is now a large number of free and commercial peak-calling software packages. Peak-calling algorithms look for 'peaks' - regions of significant tag enrichment that are typically assumed to reflect transcription factor binding to the region. While some packages simply aggregate mapped tags without regard to strand, others use strand information to locate the peaks more sensitively. Some peak-calling algorithms require the user to supply a control library whereas others can work without one, but there are several known sources of bias in sequencing reads with ChIP-seq, so that the estimation of confidence in the peaks without a control library is highly unreliable and should be avoided [6]. Confidence in the peaks is quantified using measures such as *P*-value or false discovery rate (FDR), typically based on comparisons of the ChIP library and the control library, though different peak-calling packages differ in exactly how this is done.

Some publicly available peak-calling algorithms are listed in Table 1 and several excellent and detailed reviews are available [7-9], although differences in performance between peak-callers are not well understood [9,10]. Other packages not listed in the table include GLITR, USeq, QuEST, CisGenome, Vancouver Short Read Analysis Package, spp, CCAT, ERANGE and ZINBA. Many commercial software packages also contain peak-calling functionality.

**Table 1:** Peak-calling algorithms for ChIP-seq

Name of algorithm	Notable features
MACS [23]	Uses both a control library and local statistics to minimize bias
SICER [14]	Designed for detecting diffusely enriched regions; for example, histone modification
PeakSeq [24]	Corrects for reference genome mappability and local statistics
SISSRs [25]	High resolution, precise identification of binding-site location
F-seq [26]	Uses kernel density estimation

## What are the sources of bias in the sequencing reads that you mentioned?

Many kinds of systematic biases have been described in next-generation sequencing in general and ChIP-seq in particular. A preference for sequencing C+G rich regions has been found for some platforms [11]. Mapping bias results from the frequency of occurrence of particular short homologous sequences in the genome, and from genomic amplifications and repeats. Hence the need for a control library, commonly generated by sequencing input DNA (non-ChIP genomic DNA). However, certain biases seem to remain even in the control library; in particular, genomic landmarks such as transcription start sites tend to have higher read counts even in control libraries [12]. Chromatin structure also introduces biases into the physical manipulation of DNA in ChIP experiments as a result of non-uniform shearing [13]. Specifically, silenced chromatin is harder to shear than euchromatin and will thus be underrepresented in sequence reads. So regions in transcribed genes appear to be more represented than in silent genes. Some protocols use a PCR step, which may lead to the spurious replication of reads. Therefore, most workflows filter out multiple identical copies of reads.

All mapping algorithms seek to normalize the background in such a way as to reduce the bias in reporting. As we have already said, the best approach is to have an input DNA control from cells being studied, although some protocols seek internal normalization by a sampling strategy. In cancer cell lines, regions of gene amplification can pose a further problem. False-positive peak calls are common in amplified regions simply because those regions are overrepresented in the genomic DNA sample. Amplified regions can be 'flagged' and the read counts can then be normalized to the estimated copy number. However, unless the sample has been sequenced very deeply, high sampling noise in reads from these regions - for both ChIP and control libraries - may yield unreliable estimates for the copy number and subsequently unreliable normalized values. Thus, even normalization will not be sufficient to reduce the false positives to a baseline level. While this may be acceptable if discovery of individual binding sites (followed by experimental validation) is the goal, using whole-genome binding sites in order to build a sequence-based model of transcription factor binding may require complete masking of amplified regions in the model building to reduce the effect of noisy input data.

## When do you know a ChIP-seq is not working?

If there is a control library, a ChIP-seq that is not working should result in few called peaks, and side-by-side inspection of selected genomic loci in the ChIP and control libraries should show poor enrichment. However, even when two identical libraries are sequenced, there will be several areas that may show significant count differences (as part of an FDR). The ultimate test would be the quantitative PCR validation of selected ChIP-seq peaks. For some transcription factors with well characterized motifs it can make sense to check for the occurrence of the motif in a significant fraction of the called peaks.

## You said ChIP-seq could be used for genomic analysis of histone modifications - but surely that can't be done by mapping short sequences?

It is true that most peak-calling algorithms are designed with transcription factors in mind, and such factors usually bind to short sequence elements (on the order of 10 bp). Histone marks are sometimes diffusely enriched over several nucleosomes (hundreds of base pairs) or in some cases thousands or tens of thousands of base pairs. This means that peaks may be over-called in a histone-modification-enriched region (that is, the algorithm calls several peaks where a human would prefer to view the whole region as an enriched unit) or the algorithm may fail to detect an enriched region where there is a subtle but consistent enrichment but where no single locus is enriched enough to count as a 'peak' according to the algorithm's criteria. There may also be apparent gaps in regions that are actually enriched, as a result of insufficiently deep sequencing. To avoid this, the parameters for peak-calling must be appropriately tuned.

How to do the tuning depends on the intended application. Sometimes it may be enough to compute correlation statistics for read counts with genomic landmarks such as genes, or to calculate average tag-density profiles around a set of such landmarks. If a precise demarcation of the histone-mark-enriched regions is needed, one could use a peak-calling package with explicit support for longer and more diffuse enriched regions, such as SICER [14] or CCAT [15].

## How do you know when you have sequenced enough?

The basic question is whether a library has reached the asymptotic saturation point beyond which no new binding sites will be discovered. One can try to estimate binding saturation by simulation. By running a peak-calling algorithm on successively smaller random subsets of the set of sequence reads, the number of detected peaks (on the *y *axis) can be plotted against the number of reads (on the *x *axis). This will often (but not always) result in a curve that rises rapidly in the beginning but then starts to saturate. The curve can be extrapolated to estimate at what number of sequenced reads it will start to appear flat. Estimating the exact saturation point in this way may not be possible in a strict sense, but it is usually enough to get an approximation. Obviously, a factor that binds more diffusely, such as some histone marks, will need more sequence to reach saturation. A curious observation is that some DNA-binding factors (such as RNA polymerase II) have clear saturation characteristics but for others saturation is less obvious. Although the exact reason for this is unclear, it may be that there are two populations of binding sites, one with high affinity and a second with lower affinity and greater recognition sequence degeneracy that is therefore more abundant in the genome. More sequencing will primarily uncover more sites of the lower-affinity class. Thus, for practical purposes, it may be more realistic to aim to predict the number of tags required to saturate the detection of peaks above a given target enrichment ratio (minimal enrichment saturation ratio, MSER) [16].

## Can one library be compared quantitatively with another on a site-by-site basis?

Often it is desirable to assess changes in transcription factor binding on a genomic scale over time or after ligand activation as in the case of nuclear hormone receptors. To accomplish this, multiple ChIP-seqs will need to be performed over time and the quantification of transcription factor occupancy at each site compared. In theory one should be able to compare two libraries side by side. However, one should keep in mind the biases that can give rise to differences between the libraries. These include differences in DNA fragmentation protocol, time of cross-linking, the sequencing platform, and the software and parameters used in mapping. Pre-processing steps, such as removing identical reads and amplified regions (see above), must also be done in a consistent way [17]. Finally, the depth of the sequencing reads needs to be comparable as tag counts at each peak and even the number of peaks will be proportional to the total tags sequenced.

## What can be learned using ChIP-seq?

A concrete contribution has been the identification of new regulatory elements - for example, new tissue-specific enhancers have been identified using p300-binding sites in the mouse brain [18]. ChIP-seq studies on histone modifications [1,19] have yielded insights into the functional organization of the genome on a scale that was previously unattainable. Using the genome-wide information about functional domains as defined by histone modifications, Guttman *et al*. [20] predicted and validated many large non-coding RNAs.

Perhaps the most important contribution of ChIP-seq approaches, however, is in providing a 'population' analysis of protein-DNA interactions on a genomic scale. This has shown how individual transcription factors employ different mechanisms for gene regulation depending on the degeneracy of the binding-site recognition motif, the presence of other co-localized transcription factors, and the distance from the transcription start site. In many cases the mechanism of gene regulation by a given transcription factor is specific to each particular binding site. Only through the analysis of the entire range of binding sites in the genome could some higher functional principles be discerned. As an example, ChIP-seq profiling of 13 transcription factors in embryonic stem (ES) cell development revealed the organization of regulatory elements into 'enhanceosomes' [21]. This information provided insights in the integration of transcription factor-mediated signaling pathways in ES cell differentiation.

Finally, we recently used a modification of ChIP-seq called chromatin-interaction analysis using pair end tag sequencing (ChIA-PET), in which all chromatin interactions between estrogen receptor binding sites in the genome could be identified [22]. This three-dimensional chromatin interaction map suggested that DNA topology might play a significant role in transcriptional regulation.

## What more can we expect of ChIP-seq?

Criteria for quality of experimentation will shift as understanding of the power and the limitations of a technology mature. Moreover, the depth, detail and breadth of the analysis will depend on the scientific question being asked. However, given what we now know, we can project what might be the new thresholds of acceptable experimental evidence as we go forward. First, are the antibodies used for ChIP-seq specific? We understand that the dynamics of binding will shift according to the abundance of the primary DNA-binding protein and with its cofactors. So, the specific biochemical 'states', which include the levels of transcription factors of interest, will need to be taken into account in comparisons of different cell lines. There will be greater emphasis on the overlay of binding-site maps of multiple DNA-binding proteins to provide a more comprehensive picture of interactions and complex formation.
